# Climate of the Northwest and Nervous Diseases

**Published:** 1871-10

**Authors:** 


Climate of the North-West and Nervous Diseases.—Dr. Staples in the *Northwestern Medical and Surgical Journal*, gives his views of the effects of the climate of Minnesota upon the nervous system. As compared with New England climate, that of Minnesota is tonic and *stimulating*, while that of New England is simply tonic. The climate of Minnesota is dryer, and hence its heat conducting power is less. This lack of moisture modifies its electrical condition as well as the character of light. The dryer air contains more ozone or concentrated oxygen. The moisture refracts the light as it passes through the air. Its comparative absence permits the light to come more directly to the eye, and so renders light more intense and stimulating. This condition of things has done good in some cases of consumption and harm in others. Since neuralgia is an affection of general debility and irritability of the nervous system or special nerves, a constant stimulating influence to the nerve centres can only increase the irritability without increasing the power, and this may be sufficient to destroy the nerve centre. Dr. Galloway says, "exaltation and depression are alike more marked here than at the east. Nutrition is more active and waste of tissue more rapid. The very great absorbing power of our air tends to exhilaration of nervous power, while sudden electrical change probably tends to disturb the nervous functions. Extremes are brought near, and as the intermediate grounds have to be traveled rapidly, and frequently, accidents are proportionately common. Our climate favors the production of most if not all the affections relating to the cerebro-spinal system. I think those persons whose nervous constitutions stand the climate stimulus are more robust, more powerful and more free from disease than they would be in a more humid climate. Nervous diseases are most frequent in the latter half of winter and in early spring. The different forms occur in order of frequency about as follows: sciatic, dorso-intercostal, lumbar-abdominal, cervico-occipital, trifacial, crural. The so-called *irritable heart*, I regard as a neuralgic affection of the pneumogastic nerve." Quinine as a nerve tonic has often afforded relief. In anæmic cases iron is to be combined with the quinine. Opiates, etc., afford the means of temporary relief. But severe and obstinate cases can be cured only by removing to a less stimulating or moister climate."

